# MicroRNA-5195-3p enhances the chemosensitivity of triple-negative breast cancer to paclitaxel by downregulating EIF4A2

**DOI:** 10.1186/s11658-019-0168-7

**Published:** 2019-07-01

**Authors:** Mei Liu, Can Gong, Renyuan Xu, Yu Chen, Xiaodong Wang

**Affiliations:** Department of Breast Surgery, West China Hospital of Sichuan University, 37 Guoxue Lane, Wuhou District, Chengdu, 610041 Sichuan Province China

**Keywords:** Triple-negative breast cancer, miR-5195-3p, EIF4A2, Chemosensitivity

## Abstract

**Background:**

Chemotherapy based on paclitaxel (PTX) is the standard treatment for a range of cancers, including triple-negative breast cancer (TNBC), but the increasing development of resistance has reduced/has negatively impacted its clinical utility. A previous study demonstrated that miR-5195-3p could suppress lung cancer cell growth. This study was designed to investigate whether miR-5195-3p attenuates chemoresistance to PTX by regulating target genes in TNBC cells.

**Methods:**

The study used both PTX-resistant tumor tissues and PTX-resistant TNBC cell lines. The expression of miR-5195-3p was determined using quantitative real-time PCR. Cell viability, cell cycle distribution and apoptosis were analyzed using CCK-8 and flow cytometry assays. The target genes of miR-5195-3p were predicted with bioinformatics analysis and confirmed using the luciferase reporter assay.

**Results:**

MiR-5195-3p expression was lower in PTX-resistant tumor tissues and PTX-resistant TNBC cell lines. Upregulation of miR-5195-3p enhanced the sensitivity of PTX-resistant TNBC cells to PTX treatment. EIF4A2 was confirmed as a potential target of miR-5195-3p. EIF4A2 knockdown imitated the effects of miR-5195-3p on chemosensitivity, while restoration of EIF4A2 rescued them.

**Conclusion:**

These data demonstrate that miR-5195-3p might be a potential therapeutic target to reverse chemoresistance in TNBC through its targeting of EIF4A2.

## Background

Breast cancer is the leading cause of cancer-related death among women worldwide [1]. Accounting for approximately 20% of all cases, triple negative breast cancer (TNBC) is characterized by tumors lacking epidermal growth factor receptor 2 (HER2), estrogen receptor (ER) and progesterone receptor (PR) [2]. TNBC shows more aggressive clinical behavior and poorer prognosis than other types of breast cancer [3, 4].

Paclitaxel (PTX), a chemotherapeutic agent, is commonly used for tumor therapies, including TNBC, but the increasing development of chemoresistance has negatively impacted its clinical utility for TNBC treatment [5, 6]. There is an urgent need to explore the molecular mechanisms underlying PTC chemoresistance in TNBC.

MicroRNAs (miRNAs), identified as small noncoding RNAs with 19–22 nucleotides, play a vital role in regulating gene expression. They bind to the 3′-UTR of target mRNAs causing mRNA degradation and inhibiting translation [7, 8].

There is an increasing body of evidence indicating that miRNAs function as key regulators in the development of chemoresistance. For example, upregulation of miR-383-5p significantly elevates chemosensitivity in ovarian cancer cells by targeting TRIM27 [9]. MiR-22 functions as a tumor suppressor, increasing PTX sensitivity by targeting NRAS in breast cancer cells [10]. MiR-5195-3p, a relatively recently discovered and poorly studied miRNA, has been reported to suppress cell proliferation and invasion by directing targeting the oncogene KLF5 in bladder cancer [11]. Yang et al. and Soltani et al. respectively demonstrated that miR-5195-3p exerts tumor suppressive activity in non-small cell lung cancer [12] and colon cancer [13]. Here, we are focusing on the expression and biological functions of miR-5195-3p in PTX resistance in TNBC, which has yet to be studied.

Eukaryotic translation initiation factor 4 alpha 2 (EIF4A2) is an ATP-dependent RNA helicase expressed widely in human tissues [14]. It was identified as gene biomarker of rheumatoid arthritis related to the upregulation of cell proliferation [15]. Recent articles have suggested that dysregulated expression of EIF4A2 significantly correlates to several types of cancer, including non-small cell lung cancer [16] and malignant peripheral nerve sheath tumors [17]. We speculated that EIF4A2 might be a potential oncogene affecting chemoresistance in TNBC.

In this study, we determined the expression of miR-5195-3p in PTX-resistant tumor tissues and TNBC cell lines and explored its potential function in the sensitivity of TNBC cells to PTX. We also tested our hypothesis that miR-5195-3p might participate in regulating TNBC sensitivity to PTX by regulating EIF4A2.

## Materials and methods

### Clinical tissues and cell lines

The study cohort consisted of 36 breast cancer patients who were receiving PTX-based chemotherapy at the Department of Breast Surgery in the West China Hospital of Sichuan University. The breast cancer in 18 patients showed PTX sensitivity, and in the other 18, it showed PTX resistance. Tumor tissues were collected and rapidly frozen in liquid nitrogen at − 80 °C. Before enrollment, all patients signed informed written consent. This study was approved by the Research Ethics Committee of West China Hospital of Sichuan University.

We obtained four breast cancer cell lines, including MCF-7 and three TNBC cell lines (BT549, MDA-MB-468 and MDA-MB-231), and a normal human breast epithelial cell line (MCF-10A) from the American Tissue Culture Collection (ATCC). All cell lines were cultured in ATCC-formulated Roswell Park Memorial Institute 1640 medium (RPMI-1640; Gibco-BRL) with 10% fetal bovine serum (FBS; Gibco) and maintained in a humidified incubator containing 5% CO_2_ at 37 °C.

### Establishment of PTX-resistant cell lines

PTX with a purity of at least 98% was purchased Sigma-Aldrich and reconstituted in distilled water at the appropriate concentrations. Based on a previous report [18], BT549 and MDA-MB-231 cells were exposed to continuous increasing concentrations of paclitaxel at an initial concentration of 0.5 nM over a period of 3 months. The constructed PTX-resistant cells lines that grew under this high concentration of PTX were respectively named BT549/PTX and MDA-MB-231/PTX. BT549/PTX and MDA-MB-231/PTX cells were maintained in 1 μg/ml PTX to maintain this drug-resistant phenotype.

### Cell transfection

MiR-5195-3p mimics (mimics), small interfering RNA targeting EIF4A2 (siEIF4A2) and their corresponding negative controls (miR-NC, siNC, respectively) were synthesized by GenePharma Company. In addition, the open reading frame of EIF4A2 was generated using PCR and cloned into the pcDNA 3.1 vector (Invitrogen) to generate pcDNA 3.1-EIF4A2 plasmid (EIF4A2).

For miR-5195-3p overexpression, BT549/PTX and MDA-MB-231/PTX cells were transfected with mimics or miR-NC at a final concentration of 200 nM. For EIF4A2 knockdown, BT549/PTX cells were transfected with siEIF4A2 or siNC. In the rescue experiments, EIF4A2 (2 μg/ml) was transfected into miR-5195-3p-overexpressing BT549/PTX cells. All transfections were accomplished using Lipofectamine 2000 (Invitrogen) according to the manufacturer’s instructions.

### Quantitative real-time PCR analysis

Total RNA was extracted from tissues or cells using TRIzol reagent (Takara) and cDNA was synthesized using a miScript II RT Kit or PrimeScript cDNA Synthesis Kit (Takara). Quantitative real-time PCR was performed using a miScript SYBR Green PCR Kit (Qiagen) or a LightCycler 480 SYBR Green I Master PCR Kit (Roche) for miR-5195-3p or EIF4A2 detection. The primer sequences synthesized by Sangon Biotech (Shanghai) were: miR-5195-3p, forward: 5′-TAGCAGACTCTTATGATG-3′ and reverse: 5′-TGGTGGAGTCGTCGTG-3′; U6, forward: 5′-CTCGCTTCGGCAGCACA-3′ and reverse: 5′-AACGCTTCACGAATTTGCGT-3′; EIF4A2 forward: 5′-ATGTTCGCTTCGAGACGTGC-3′; and reverse: 5′-TTTCACTAAAGGCAGAGAGTCAG-3′; and GAPDH forward: 5′-ATCGTCCACCGCAAATGCTTCTA-3′; and reverse: 5′-AGCCATGCCAATCTCATCTTGTT-3′. The relative expressions of miR-5195-3p or EIF4A2 were respectively normalized to the expression of U6 or GAPDH.

### Paclitaxel sensitivity assay

The CCK-8 assay was performed to assess cell sensitivity to paclitaxel. Briefly, cells from different groups were seeded in 96-well plates at a density of 1.5 × 10^4^ cells per well and incubated with serially diluted paclitaxel (0, 2, 4, 6 and 8 μg/ml), followed by 2 h incubation with CCK-8 solution (Sigma-Aldrich). The absorbance of each well was measured at a wavelength of 450 nm using a microplate reader (Bio-Rad). The cell survival rate was calculated based on the absorbance of the control cells. IC_50_ values were determined as the drug concentration causing 50% cell growth inhibition.

### Flow cytometry analysis

Cells from different groups were digested with trypsin, washed three times with cold phosphate-buffered saline (PBS) and fixed with 80% ethanol overnight at 4 °C. After centrifugation, the cells were washed twice with cold PBS.

The cells were stained with propidium iodide (PI, Nanjing KeyGen Biotech) for 30 min at 37 °C for cell cycle analysis or Annexin V-fluorescein isothiocyanate (FITC) and PI (Nanjing KeyGen Biotech) for apoptosis analysis. A flow cytometer equipped with FlowJo 10 software (BD Biosciences) was used to determine cell cycle distribution and apoptosis rate.

### Prediction of miRNA target and luciferase reporter assay

The target genes of miR-5195-3p were predicted using the minRNA database (TargetScan and miRBase). Based on the cellular function assay, EIF4A2 was selected as potential target gene of miR-5195-3p and assessed using the luciferase reporter assay. Briefly, the 3′-UTR of EIF4A2 mRNA was synthesized and cloned into a psiCHECK-reporter plasmid (Promega) and then validated through DNA sequencing. This was named wide-type EIF4A2 (WT EIF4A2). Using QuikChange Multi Site-Directed Mutagenesis kit (Stratagene), mutant EIF4A2 (MUT EIF4A2) plasmid was generated according to the manufacturer’s protocol. Next, BT549/PTX and MDA-MB-231/PTX cells were co-transfected with recombinant reporter WT or MUT plasmids and the mimics or miR-NC in 24-well plates. After 48 h, the luciferase activities were measured using the Dual Luciferase Reporter Assay System (Promega).

### Western blotting

Total protein was extracted from cells using cell lysis buffer and the concentration was determined using a BCA assay kit (Beyotime Biotechnology). Approximately 30 μg of total protein was separated using 10% SDS-PAGE and transferred onto polyvinylidene difluoride (PVDF) membranes (GE Healthcare). The membrane was blocked overnight in 5% non-fat milk in TBST (Sigma-Aldrich), then incubated with primary antibodies against EIF4A2 and GAPDH at room temperature for 2 h, followed by incubation with horseradish peroxidase-labeled secondary antibody. Protein bands were evaluated using an enhanced chemiluminescence detection system (Bio-Rad).

### Statistical analysis

All data were analyzed using SPSS 17.0 and expressed as means ± SD. Differences between groups were analyzed using Student’s t test or one-way analysis of variance, followed by the Tukey-Kramer post hoc test. Correlation between EIF4A2 mRNA and miR-5195-3p was estimated using Spearman’s correlation method. *p* < 0.05 was considered significant.

## Results

### MiR-5195-3p was downregulated in PTX-resistant breast cancer tissue and cells

**Fig. 1:**
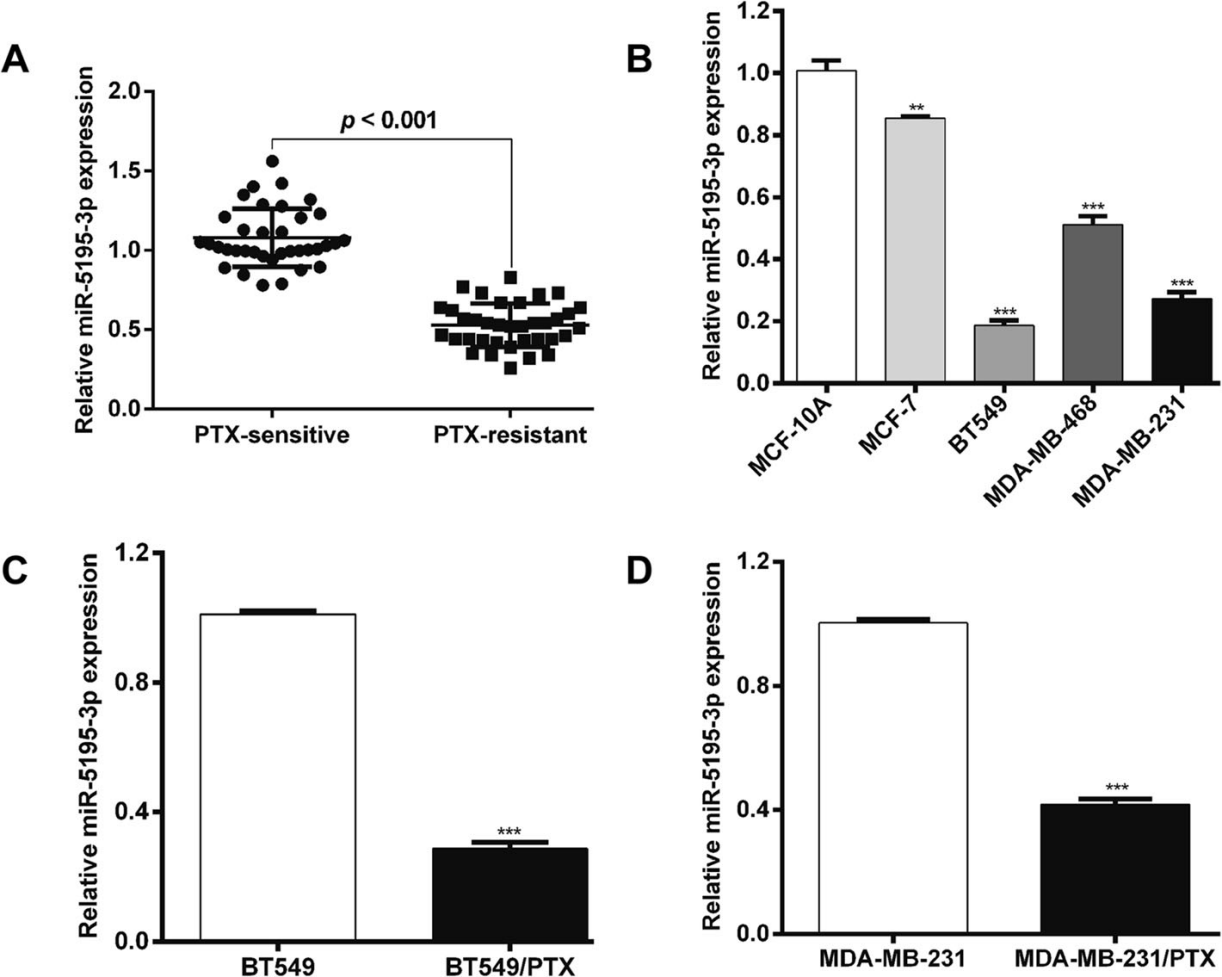
MiR-5195-3p was downregulated in paclitaxel-resistant breast cancer tissue and cells. **a** Using quantitative real-time PCR, the expression of miR-5195-3p was determined in breast cancer samples from 36 patients: 18 with paclitaxel-sensitive tumors and 18 with paclitaxel-resistant ones. **b** The expression of miR-5195-3p was determined in the indicated breast cancer cell lines and in the normal breast epithelial cell line (MCF-10A). **p < 0.01, ***p < 0.001 vs. MCF-10A. **c** and **d** The expression of miR-5195-3p was determined in paclitaxel-resistant BT549 and MDA-MB-231 cells and the corresponding parental cell lines. ***p < 0.001 vs. BT549 or MDA-MB-231

To verify the expression level of miR-5195-3p in PTX-resistant breast cancer tissue, quantitative real-time PCR was performed with samples from 36 patients: 18 with PTX-sensitive and 18 with PTX-resistant tumors. MiR-5195-3p was significantly downregulated in PTX-resistant breast cancer tissues compared with its level in PTX-sensitive tissues (Fig. 1a). We found the miR-5195-3p level was significantly lower in all four breast cancer cell lines (MCF-7, BT549, MDA-MB-468 and MDA-MB-231) than in the normal breast epithelial cell line, MCF-10A. Notably, the lowest level of miR-5195-3p was seen in two of the TNBC cell lines, BT549 and MDA-MB-231 (Fig. 1b). In the constructed PTX-resistant cells BT549/PTX and MDA-MB-231/PTX, we found that the miR-5195-3p level was significantly lower (Fig. 1c and d) than in the corresponding parental cells. These results show that miR-5195-3p is downregulated in PTX-resistant breast cancer tissue and cells, which might be an important molecular role in chemoresistance.

### Upregulated miR-5195-3p activated the PTX sensitivity of TNBC cells

**Fig. 2:**
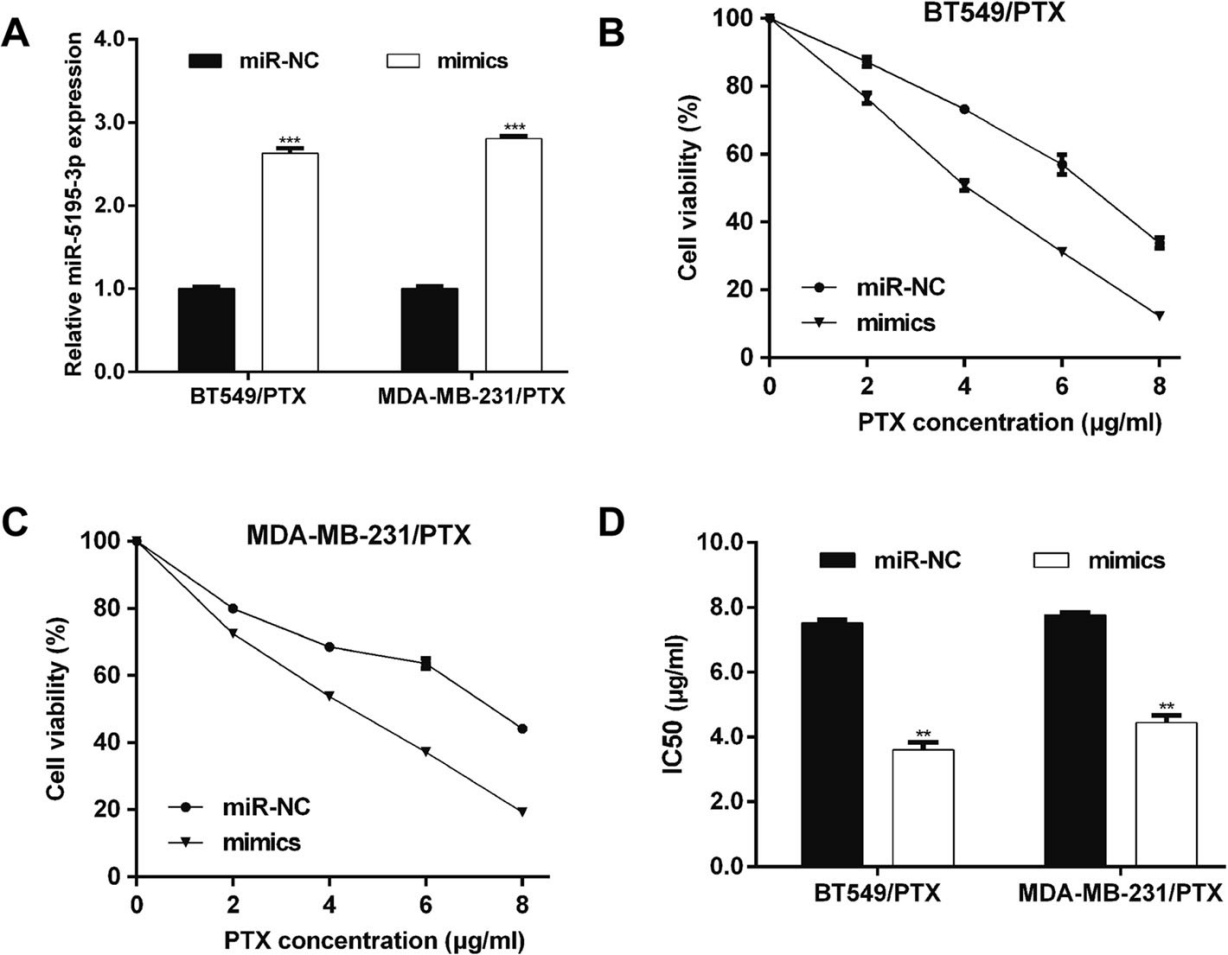
MiR-5195-3p enhanced the paclitaxel sensitivity of breast cancer cells. **a** Synthesized miR-5195-3p mimics were transfected into paclitaxel-resistant MCF-7/PTX and MDA-MB-231/PTX cells. MiR-5195-3p expression was measured using quantitative real-time PCR. **b** and **c** CCK-8 assay was performed to determine the cell sensitivity to paclitaxel of MCF-7/PTX and MDA-MB-231/PTX cells. **d** The 50% inhibitory concentration (IC_50_) value of paclitaxel in MCF-7/PTX and MDA-MB-231/PTX cells transfected with mimics or miR-NC. ***p < 0.001 vs. miR-NC

We synthesized and transfected mimics to upregulate the miR-5195-3p expression levels in BT549/PTX and MDA-MB-231/PTX cells, as confirmed using quantitative real-time PCR analysis (Fig. 2a). The CCK-8 assay showed that the cell survival rate was obviously suppressed in both BT549/PTX (Fig. 2b) and MDA-MB-231/PTX cells (Fig. 2c) after treatment with various doses of PTX (0, 2, 4, 6 and 8 μg/ml). In addition, the IC_50_ values for PTX in BT549/PTX and MDA-MB-231/PTX cells were significantly lower in the mimic groups than in the miR-NC group (Fig. 2d).

**Fig. 3:**
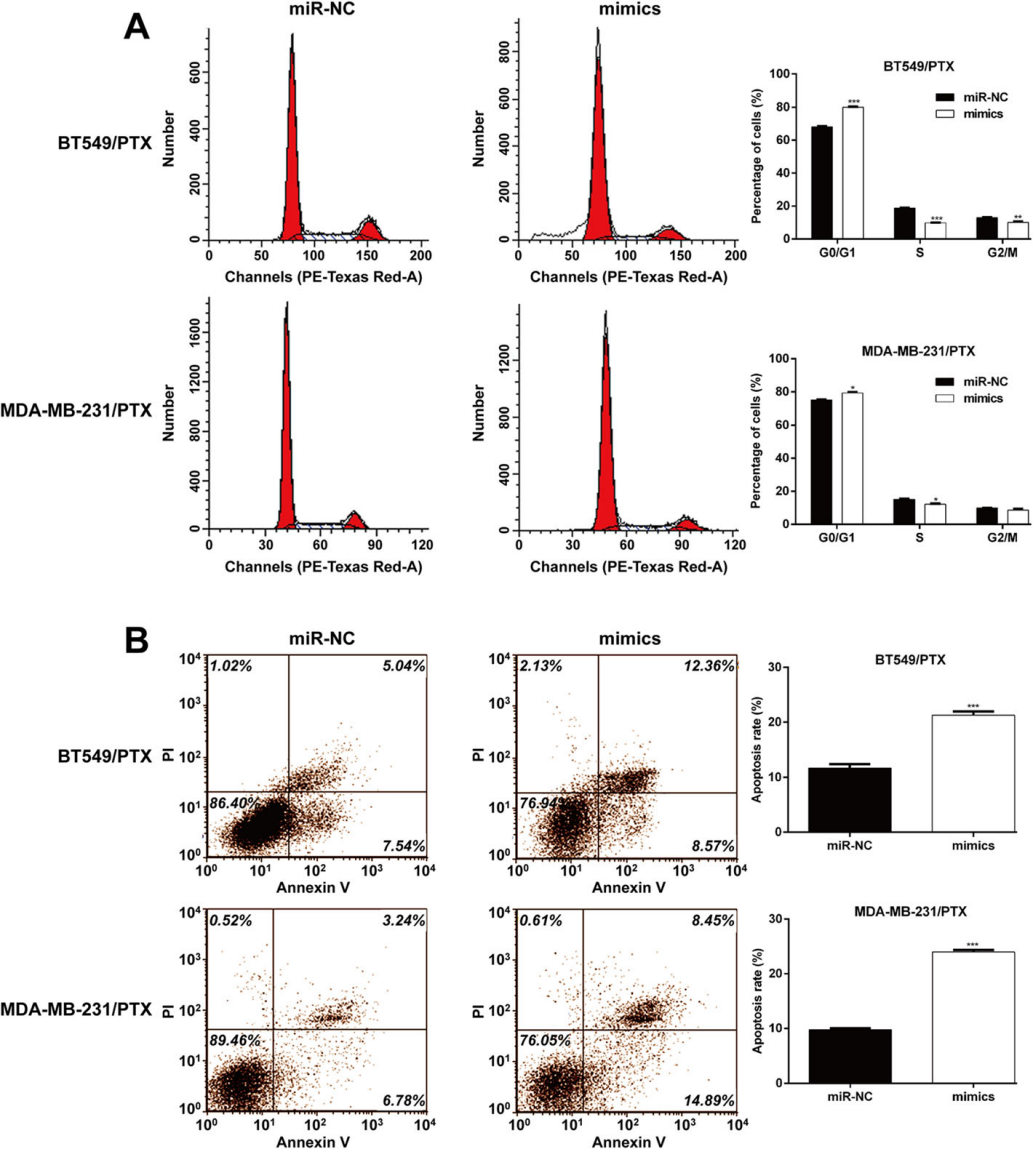
MiR-5195-3p induced cell cycle arrest and apoptosis of paclitaxel-resistant breast cancer cells. Synthesized miR-5195-3p mimics were transfected into paclitaxel-resistant MCF-7/PTX and MDA-MB-231/PTX cells. **a** Representative images of cell cycle distribution are shown in left panel and the statistical analysis of the cell percentage in G0/G1, S and G2/M phases is shown in the right panel. **b** Representative images of apoptosis are shown in left panel and the statistical analysis of apoptosis is shown in the right panel. *p < 0.05, **p < 0.01, ***p < 0.001 vs. miR-NC

These data indicate that the PTX sensitivity of TNBC cells was elevated upon miR-5195-3p overexpression. Moreover, flow cytometry with the PI staining assay (Fig. 3a) showed that the cell distribution was significantly higher at G0/G1 phase in the miR-5195-3p-overexpressing group than in the miR-NC group in both BT549/PTX and MDA-MB-231/PTX. Flow cytometry with the Annexin V/PI double staining assay (Fig. 3b) revealed that miR-5195-3p mimic transfection significantly promoted cell apoptosis in BT549/PTX and MDA-MB-231/PTX cells, in comparison with the results for the miR-NC transfection. These findings suggest that miR-5195-3p could significantly elevate the PTX sensitivity of TNBC cells in vitro.

### EIF4A2 is a direct target of miR-5195-3p

**Fig. 4:**
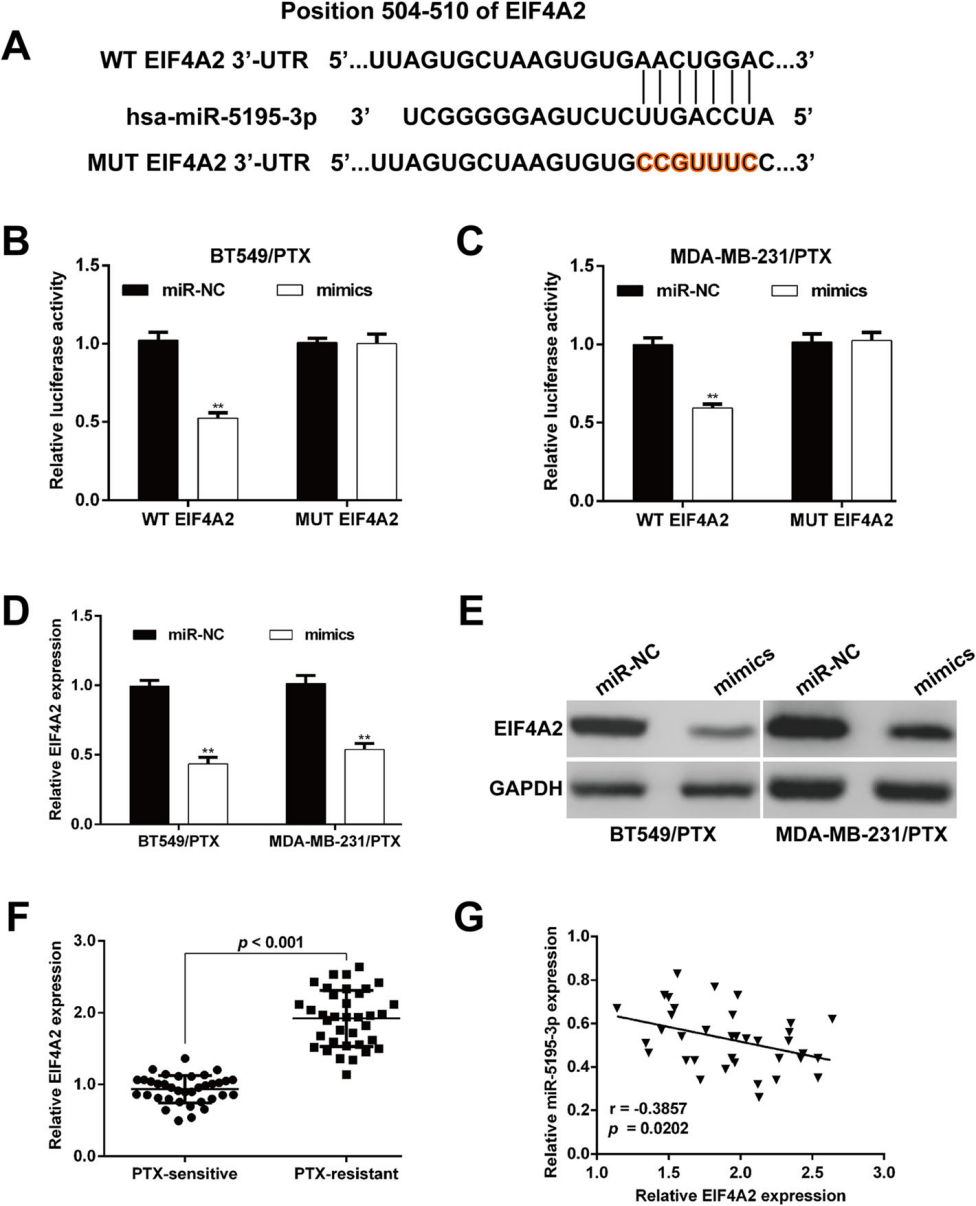
EIF4A2 is a direct target of miR-5195-3p. **a** The binding sites of EIF4A2 and miR-5195-3p were predicted using bioinformatics analysis. **b** and **c** The luciferase reporter assay was performed in paclitaxel-resistant BT549/PTX and MDA-MB-231/PTX cells. **d** and **e** Quantitative real-time PCR and western blot assays were used to analyze the expression of EIF4A2 after transfection of miR-5195-3p mimics and miR-NC. **f** The expression of EIF4A2 was determined via quantitative real-time PCR in 18 paclitaxel-sensitive and 18 paclitaxel-resistant tissues. **g** Spearman rank correlation analysis showed an inverse correlations between the expression levels of EIF4A2 and miR-5195-3p in paclitaxel-resistant breast cancer tissues. **p < 0.01 vs. miR-NC

We used TargetScan and miRBase to analyze the underlying mechanism of miR-5195-3p in the PTX sensitivity of TNBC cells. Of the predicted target genes of miR-5195-3p, EIF4A2 was selected for its association with cell proliferation. Figure 4a shows that the 3′-UTR regions of EIF4A2 contained the binding site for the miR-5195-3p seed region. Subsequently, WT EIF4A2 or MUT EIF4A2 reporter plasmids were transfected into PTC-resistant TNBC cell lines together with mimics or miR-NC. We found that ectopic expression of miR-5195-3p significantly decreased the luciferase activity of WT EIF4A2 3′-UTR in both BT549/PTX (Fig. 4b) and MDA-MB-231/PTX cells (Fig. 4c), in comparison with the results with miR-NC. These suppressive effects were abolished with a mutated miR-5195-3p binding site for EIF4A2. What’s more, quantitative real-time PCR (Fig. 4d) and western blot analysis (Fig. 4e) showed that EIF4A2 expression was downregulated in miR-5195-3p-overexpressing BT549/PTX and MDA-MB-231/PTX cells.

We next measured the EIF4A2 mRNA expression level in PTX-resistant breast cancer tissues and found that it was significantly higher in PTX-resistant breast cancer tissues than in PTX-sensitive samples (Fig. 4f). Pearson correlation analysis further showed an inverse correlation between the levels of EIF4A2 and miR-5195-3p in PTX-resistant breast cancer tissues (Fig. 4g; *p* = 0.0202). Our results suggest that EIF4A2 might be a novel target of miR-5195-3p.

### MiR-5195-3p enhanced the PTX sensitivity of TNBC cells by downregulating EIF4A2

**Fig. 5:**
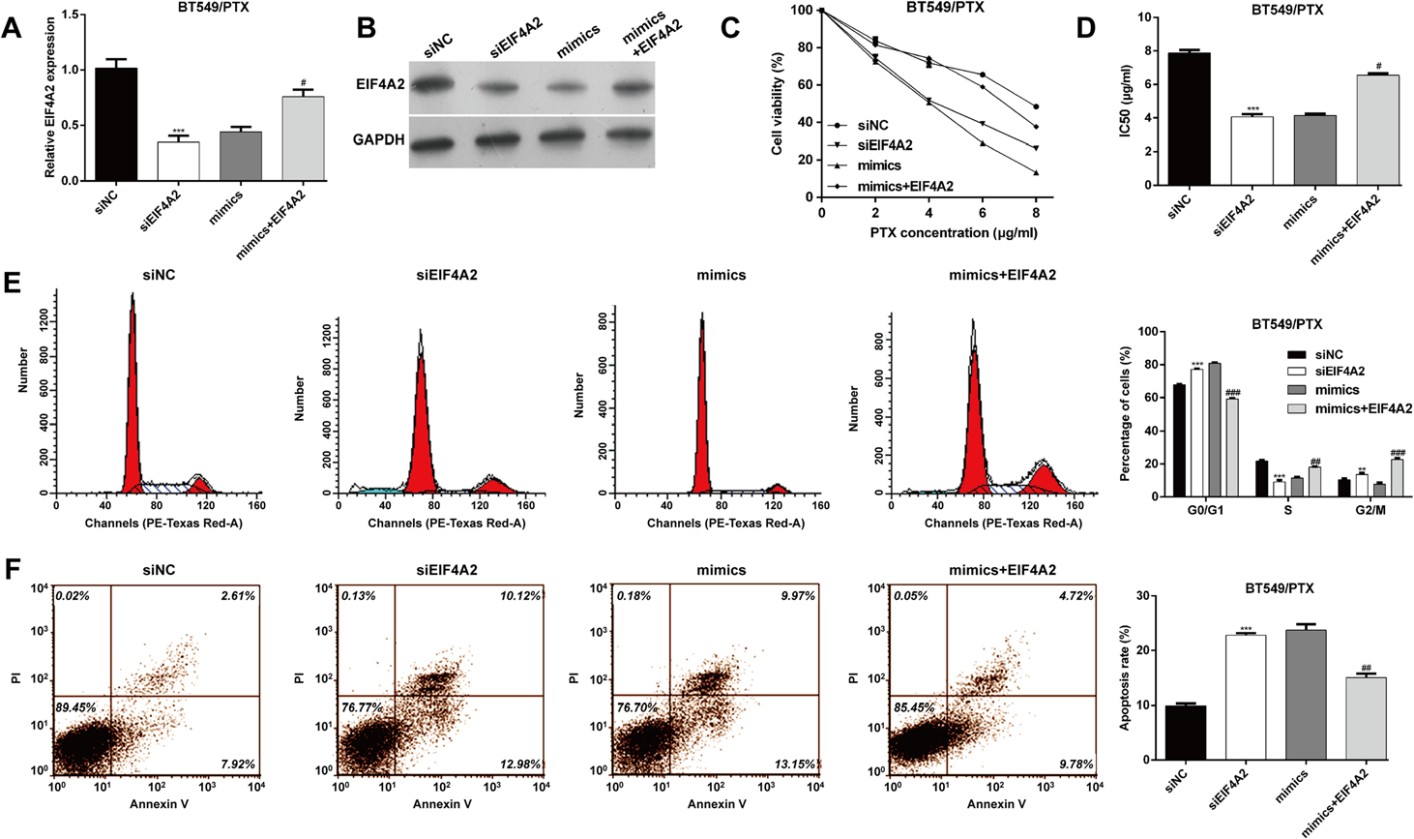
MiR-5195-3p enhanced the paclitaxel sensitivity of TNBC cells by downregulating EIF4A2. BT549/PTX cells were transfected with siNC, siEIF4A2, mimics, mimics + EIF4A2, respectively. **a** and **b** Quantitative real-time analysis and western blotting were used to determine the expression of EIF4A2 in paclitaxel-resistant BT549/PTX cells. **c** and **d** CCK-8 assay was performed to determine the IC_50_ value of paclitaxel in BT549/PTX cells from different groups. **e** Representative images of cell cycle distribution are shown in the left panel and a statistical analysis of the cell percentage in G0/G1, S and G2/M phases is shown in the right panel. **f** Representative images of apoptosis are shown in the left panel and a statistical analysis of apoptosis is shown in the right panel. ***p < 0.001 vs. siNC; ^#^*p* < 0.05, ^##^*p* < 0.01, ^###^*p* < 0.001 vs. mimics

To explore whether miR-5195-3p mediated chemosensitivity by targeting EIF4A2 in PTX-resistant cells, BT549/PTX cells were transfected with siNC, siEIF4A2, mimics or mimcs + EIF4A2. Quantitative real-time PCR (Fig. 5a) and western blot analysis (Fig. 5b) confirmed that the expression of EIF4A2 significantly decreased after EIF4A2 knockdown, but its upregulation was partially attenuated after EIF4A2 overexpression. The in vitro functional experiments further indicated that transfection with siEIF4A2 significantly reduced the IC_50_ value compared with siNC transfection, and induced G0/G1 phase arrest and apoptosis. More importantly, EIF4A2 overexpression abrogated the effects of miR-5195-3p overexpression on the IC_50_ value, G0/G1 cell cycle arrest and apoptosis, as assessed with CCK-8 (Fig. 5c and d), flow cytometry with PI (Fig. 5e) and flow cytometry with Annexin V/PI staining (Fig. 5f). These findings indicate that miR-5195-3p enhanced the PTX sensitivity of TNBC cells by downregulating EIF4A2.

## Discussion

Chemoresistance is the main obstacle in cancer therapy. It leads to poorer prognoses, higher recurrence and lower five-year survival rates [19, 20]. Here, we investigate the role of miR-5195-3p in PTX resistance in TNBC and explore the underlying mechanism for drug resistance. We found miR-5195-3p was downregulated in PTX-resistant breast cancer tissue and cells and demonstrated that miR-5195-3p overexpression could significantly enhance the PTX sensitivity of TNBC cells.

In recent years, miRNAs have attracted considerable attention as new players in chemoresistance in tumors. Pan et al. found that miR-503 enhances the drug sensitivity of MCF-7/ADR cells through suppression of EIF4G [21]. Chen et al. reported that miR-504 contributes to cisplatin resistance in MG63 osteosarcoma cells by suppressing p53 [22]. Previous studies implied that miR-5195-3p is downregulated in several cancers, including bladder cancer [11] and lung cancer [12]. In our study, we found that overexpression of miR-5195-3p inhibited the activity of cell proliferation and induced G0/G1 phase arrest and apoptosis in PTX-resistant TNBC cells. This could imply its tumor suppressive role in PTX-resistant TNBC cells.

Using bioinformatics tools and the luciferase reporter assay, we further identified and validated the target genes of miR-5195-3p in regulating PTX chemoresistance: EIF4A2. As expected, we found EIF4A2 was upregulated in PTX-resistant breast cancer tissues and negatively correlated with miR-5195-3p expression.

EIF4A2 was previously shown to be necessary for efficient HIV-1 replication [23]. In addition, EIF4A2 was associated with elevated cell proliferation in rheumatoid arthritis [15], non-small-cell lung cancer [16] and malignant peripheral nerve sheath tumors [17]. Consistent with this evidence, we demonstrated that EIF4A2 knockdown imitated the effects of miR-5195-3p on chemosensitivity in TNBC cells, whereas restoration of EIF4A2 levels caused chemoresistance to increase again. Garrido et al. recently demonstrated that targeting the EIF4F translation initiation complex could be a novel therapeutic strategy to eradicate taxane-resistant prostate cancer cells [24]. Considering the similarities between members of EIF the family, we speculate that EIF4A2 knockdown treatment may offer a novel strategy to combat TNBC chemoresistance.

## Conclusions

This study demonstrated the vital role of miR-5195-3p in the PTX resistance of TNBC. Our results confirm that miR-5195-3p enhances the paclitaxel sensitivity of TNBC cells by downregulating EIF4A2, which suggests a novel molecular mechanism of TNBC chemotherapy resistance. Of course, more target genes and pathways of miR-5195-3p will be explored to further elucidate the role of miR-5195-3p in chemotherapy resistance.

## Data Availability

The data from this study are available in this published article.

## Abbreviations

EIF4A2: Eukaryotic translation initiation factor 4 alpha 2
MUT: Mutant
PTX: Paclitaxel
siRNA: Small interfering RNA
TNBC: Triple negative breast cancer
WT: Wild-type

## References

[1] Yang R, Xing L, Zheng X, Sun Y, Wang X, Chen J (2019). The circRNA circAGFG1 acts as a sponge of miR-195-5p to promote triple-negative breast cancer progression through regulating CCNE1 expression. Mol Cancer.

[2] Zeng YX, Zhu J, Peng C, Liang W, Lin H (2019). Knockdown of nucleophosmin 1 suppresses proliferation of triple-negative breast cancer cells through activating CDH1/Skp2/p27kip1 pathway. Cancer Manag Res.

[3] Carey LA, Perou CM, Livasy CA, Dressler LG, Cowan D, Conway K, Karaca G, Troester MA, Tse CK, Edmiston S, Deming SL, Geradts J, Cheang MC, Nielsen TO, Moorman PG, Earp HS, Millikan RC (2006). Race, breast cancer subtypes, and survival in the Carolina breast Cancer study. JAMA.

[4] Xu YL, Yao R, Li J, Zhou YD, Mao F, Pan B, Sun Q (2017). FOXC1 overexpression is a marker of poor response to anthracycline-based adjuvant chemotherapy in sporadic triple-negative breast cancer. Cancer Chemother Pharmacol.

[5] Liao Wei-Siang, Ho Yu, Lin Yu-Wei, Naveen Raj Emmanuel, Liu Kuang-Kai, Chen Chinpiao, Zhou Xiao-Zhen, Lu Kun-Ping, Chao Jui-I (2019). Targeting EGFR of triple-negative breast cancer enhances the therapeutic efficacy of paclitaxel- and cetuximab-conjugated nanodiamond nanocomposite. Acta Biomaterialia.

[6] Kim SH, Park HJ, Moon DO (2017). Sulforaphane sensitizes human breast cancer cells to paclitaxel-induced apoptosis by downregulating the NF-κB signaling pathway. Oncol Lett.

[7] Guo H, Ingolia NT, Weissman JS, Bartel DP (2010). Mammalian microRNAs predominantly act to decrease target mRNA levels. Nature.

[8] Liu C, Su C, Chen Y, Li G (2018). MiR-144-3p promotes the tumor growth and metastasis of papillary thyroid carcinoma by targeting paired box gene 8. Cancer Cell Int.

[9] Jiang J, Xie C, Liu Y, Shi Q, Chen Y (2019). Up-regulation of miR-383-5p suppresses proliferation and enhances chemosensitivity in ovarian cancer cells by targeting TRIM27. Biomed Pharmacother.

[10] Song YK, Wang Y, Wen YY, Zhao P, Bian ZJ (2018). MicroRNA-22 Suppresses Breast Cancer Cell Growth and Increases Paclitaxel Sensitivity by Targeting NRAS. Technol Cancer Res Treat.

[11] Jiang Z, Zhang Y, Cao R, Li L, Zhong K, Chen Q, Xiao J (2017). miR-5195-3p inhibits proliferation and invasion of human bladder Cancer cells by directly Targeting oncogene KLF5. Oncol Res.

[12] Yang Q (2019). MicroRNA-5195-3p plays a suppressive role in cell proliferation, migration and invasion by targeting MYO6 in human non-small cell lung cancer. Biosci Biotechnol Biochem.

[13] Jahangiri Moez Mahnaz, Bjeije Hassan, Soltani Bahram M. (2019). Hsa-miR-5195-3P induces downregulation of TGFβR1, TGFβR2, SMAD3 and SMAD4 supporting its tumor suppressive activity in HCT116 cells. The International Journal of Biochemistry & Cell Biology.

[14] Sudo K, Takahashi E, Nakamura Isolation Y (1995). Mapping of the human EIF4A2 gene homologous to the murine protein synthesis initiation factor 4A-II gene Eif4a2. Cytogenet Cell Genet.

[15] Lu C, Niu X, Xiao C, Chen G, Zha Q, Guo H, Jiang M, Lu A (2012). Network-based gene expression biomarkers for cold and heat patterns of rheumatoid arthritis in traditional chinese medicine. Evid Based Complement Alternat Med.

[16] Shaoyan X, Juanjuan Y, Yalan T, Ping H, Jianzhong L, Qinian W (2013). Downregulation of EIF4A2 in non-small-cell lung cancer associates with poor prognosis. Clin Lung Cancer.

[17] Oblinger JL, Burns SS, Akhmametyeva EM, Huang J, Pan L, Ren Y, Shen R, Milesmarkley B, Moberly AC, Kinghorn AD (2016). Components of the eIF4F complex are potential therapeutic targets for malignant peripheral nerve sheath tumors and vestibular schwannomas. Neuro Oncol.

[18] Zhen NW, Yatim SMJM, Kohlbauer VK, Feng M, Jian YG, Yi B, Lee PL, Zhang S, Wang PP, Lim E (2015). IRAK1 is a therapeutic target that drives breast cancer metastasis and resistance to paclitaxel. Nat Commun.

[19] Yao J, Yao X, Tian T, Fu X, Wang W, Li S, Shi T, Suo A, Ruan Z, Guo H, Nan K, Huo X (2017). ABCB5-ZEB1 Axis promotes invasion and metastasis in breast Cancer cells. Oncol Res.

[20] Geretto M, Pulliero A, Rosano C, Zhabayeva D, Bersimbaev R, Izzotti A (2017). Resistance to cancer chemotherapeutic drugs is determined by pivotal microRNA regulators. Am J Cancer Res.

[21] Pan X, Yang X, Zang J, Zhang S, Huang N, Guan X, Zhang J, Wang Z, Li X, Lei X (2017). Downregulation of eIF4G by microRNA-503 enhances drug sensitivity of MCF-7/ADR cells through suppressing the expression of ABC transport proteins. Oncol Lett.

[22] Chen X, Lv C, Zhu X, Lin W, Wang L, Huang Z, Yang S, Sun J (2019). MicroRNA-504 modulates osteosarcoma cell chemoresistance to cisplatin by targeting p53. Oncol Lett.

[23] Ndzinu JK, Takeuchi H, Saito H, Yoshida T, Yamaoka S (2018). eIF4A2 is a host factor required for efficient HIV-1 replication. Microbes Infect.

[24] Garrido MF, Martin NJ, Bertrand M, Gaudin C, Commo F, El Kalaany N, Al Nakouzi N, Fazli L, Del Nery E, Camonis J, Perez F, Lerondel S, Le Pape A, Compagno D, Gleave M, Loriot Y, Desaubry L, Vagner S, Fizazi K, Chauchereau A (2019). Regulation of eIF4F Translation Initiation Complex by the Peptidyl Prolyl Isomerase FKBP7 in Taxane-resistant Prostate Cancer. Clin Cancer Res.

